# History-Based Response Threshold Model for Division of Labor in Multi-Agent Systems

**DOI:** 10.3390/s17061232

**Published:** 2017-05-28

**Authors:** Wonki Lee, DaeEun Kim

**Affiliations:** School of Electrical and Electronic Engineering, Yonsei University, 50 Yonsei-ro, Seodaemun-gu, Seoul 120-749, Korea; wonkilee@yonsei.ac.kr

**Keywords:** multi-robot system, dynamic task allocation, division of labor, response threshold model, task demand, specialization

## Abstract

Dynamic task allocation is a necessity in a group of robots. Each member should decide its own task such that it is most commensurate with its current state in the overall system. In this work, the response threshold model is applied to a dynamic foraging task. Each robot employs a task switching function based on the local task demand obtained from the surrounding environment, and no communication occurs between the robots. Each individual member has a constant-sized task demand history that reflects the global demand. In addition, it has response threshold values for all of the tasks and manages the task switching process depending on the stimuli of the task demands. The robot then determines the task to be executed to regulate the overall division of labor. This task selection induces a specialized tendency for performing a specific task and regulates the division of labor. In particular, maintaining a history of the task demands is very effective for the dynamic foraging task. Various experiments are performed using a simulation with multiple robots, and the results show that the proposed algorithm is more effective as compared to the conventional model.

## 1. Introduction

Multi-agent systems can be used to perform dynamic tasks. Several effective and adaptive behaviors have been identified in recent studies: task allocation [1,2,3], searching [4,5,6] and foraging [7,8,9]. Each member of a group of robots should determine the current task that is most commensurate with its current surrounding labor states. Generally, there are two issues involved in performing tasks in a multi-robot system: the cooperation of robots and the division of labor among robots in a group. The cooperation involves performing a complex task as a group of robots instead of improving the ability of a single robot, while the division of labor involves efficiently managing tasks that are costly and time intensive. One possible approach for solving these types of problems is to establish a dynamic task allocation mechanism using adaptive processing that independently adapts all robots in a group to a dynamically-changing environment. The basic requirements for achieving this performance are maintaining the specialized individuals and processing tasks in parallel.

Swarm intelligence has been applied to multi-agent task control algorithms. This area of research has focused on a large group of relatively simple agents that can collectively solve a complex problem. This concept is useful for designing a task allocation model in a multi-robot system. A single robot has limited hardware ability, and it has a few behavioral rules. However, a multi-robot system can effectively perform more difficult tasks because this model has the advantage of concurrency and fault tolerance. It enables both the simultaneous performance of tasks in several locations and flexibility in an individual failure in a large-scale system.

In recent works, agent-based approaches, inspired from several social insect species, have gained increasing attention as the solution to the multi-robot system problem. They can achieve effective task allocations based on the specialization tendency, i.e., different tasks are simultaneously performed at different places by specialized individuals [10]. Examples of labor division include nest defense and foraging in ants [11] and nursing [12] and nectar and pollen collection [13] in honeybees. To explain these phenomena, a response threshold model was proposed whereby each agent is assigned a given task if the stimulus of that task exceeds the corresponding response threshold value [14]. Each individual has response thresholds for multiple candidate tasks required for colony survival. An agent with a low response threshold value for a given task tends to perform that task if the stimulus is low. Conversely, an individual with a high response threshold value for a given task performs the task if the stimulus is high. The mechanism for regulating products, such as food, can be explained using this model by assigning the task of foraging to individual agents as the demand of some specific product increases.

In the response threshold model, individuals can have a specialized tendency as a strong or soft type. Usually, the strong specialization is decided on the basis of genetic factors such as age and body shape [15]. The member with the strong specialization performs only one or a few activities among all of the tasks required to be completed for the colony survival. However, unpredictable changes in the environment and the fluctuating proportion of members result in a supplementary mechanism within a group for maintaining an adaptive division of labor task allocation. Therefore, the members with the soft specialization are required to perform several activities. They usually perform the urgent task required by the group at each moment. For example, if the number of specialists of Task A decreases, the demand for Task A increases, and accordingly, some specialists of Task B perform Task A, which they would normally not do [16].

An interesting task allocation problem is the foraging task for colony survival. The foraging task has been used as a test in a multi-robot system. In a simple forging task, a group of robots must collect objects of a single type, usually for energy balancing or a similar objective. Arkin et al. (1993) [17] studied the impact of the introduction of simple communication on the performance of a society of robots in a single-prey foraging task. They used the minimal knowledge of the behavioral state of the fellow agents. A behavior-based algorithm using reinforcement learning was proposed to induce the robots to learn how to balance each of the behaviors. The objective was to optimize the performance in a foraging task [18]. Krieger and Billeter (2000) [19] used an energy level to maintain the energy stocked in their nest by collecting a food item. A similar task allocation problem motivated by energy gain has also been previously studied [20]. In recent works, self-organized task allocation has been studied to handle tasks that are both distributed and sequential [21]. In a multi-foraging task, one or multiple objects are collected in complex environments. A group of robots is used to identify an efficient multi-foraging task, whereby efficiency is defined as a function of the energy wasted while performing tasks [22] or the energy level in a nest [23]. A multi-foraging task was used to demonstrate the use of a mathematical model for a general dynamic task allocation mechanism [24].

In this paper, the response threshold model that was realized for an effective division of labor and the specialization that was inspired by insect societies are applied to handle the foraging task in a group of robots. This model has been studied for the purpose of task allocation in a swarm of robots. A fixed response threshold model using a randomly-generated threshold value was used for a foraging mission [23], and the adaptive response threshold model in which the threshold value is adaptively changed depending on the environmental circumstances has been suggested [25]. A classical foraging task in which robots decided whether they would go foraging or remain in a standby mode near the base area was performed.

Most aligned with this work is Jones and Mataric’s work (2003) [26], where the division of labor is achieved in a distributed manner. They suggest a ratio model for the division of labor. In this model, two types of objects are used (e.g., pucks), and each robot is equally capable of foraging for both puck types, but can only be allocated to forage for a single type at a time. According to the control policy, a robot may switch the foraged puck type from red to green depending on whether the ratio of red puck entries in the view history is smaller than the ratio of robot entries foraging for red pucks, and similarly, a robot may switch the foraged puck type from green to red depending on whether the ratio of green puck entries in the view history is smaller than the ratio of robot entries foraging for green pucks. The ratio of robot entries and that of puck entries that the robot has sensed are used to determine the foraging state of the robot.

Several variables could be considered for the evaluation of the performance in a foraging task. The performances can be evaluated based on the total time required to complete a given task, energy consumption, actual amount of task allocations, number of foraged objects and number of task changes. Moreover, if costs are incurred due to the switching of the current task to another, the minimization of the task switching is beneficial to the overall system performance. Simulation-based experiments were used to demonstrate the robustness and adaptability of the proposed approach to environmental variations, and the division of labor is demonstrated with minimum task switching to obtain the specialization for specific tasks.

The remainder of the paper is organized as follows. Section 2 presents related works on the topic of the foraging task, which includes the behavior of the robot. Section 3 presents the proposed approach for solving the given foraging task problem. Section 4 describes the experiments and results, and Section 5 presents the conclusions and discusses future works.

## 2. Problem Description

### 2.1. Task Scenario

In typical foraging tasks, an individual robot or robots in a group collect objects from an environment and immediately remove the objects from that spot [27] or transfer them to another common location such as a nest or transfer cache [28,29]. The foraging taxonomies [30] and multi-robot coordination problems and solutions [31] have been studied. In this paper, a dynamic task allocation problem similar to that in Lerman et al. (2006) [24], which handles the foraging problem with two types of objects—red and green pucks—according to their colors is considered.

**Figure 1:**
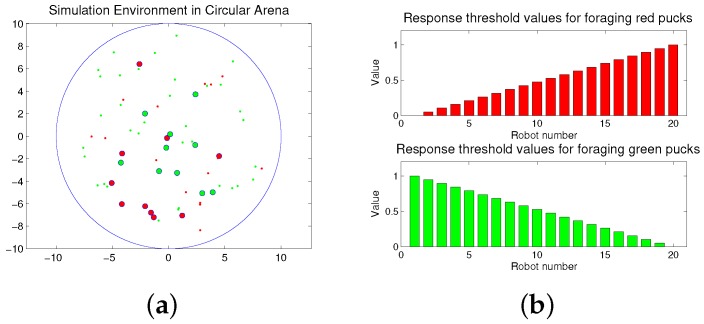
(**a**) Snapshot of the simulation environment; (**b**) response threshold values of robots for two tasks.

The foraging task is performed in a circular arena as shown in Figure 1a. The working area of the arena is 315 m^2^, and the radius is approximately 10 m. Twenty robots explore the arena to forage for 50 pucks. The large red and green circles are robots, and the small red and green dots are pucks. In the beginning, the pucks and robots are randomly distributed in the arena. The robots move to forage for the pucks during a given time span. If a robot approaches one puck, the puck is immediately removed from the environment. After a puck is removed, a new puck of the same type or the same color is placed in an arbitrary location in order to maintain a constant number of pucks in the arena, and the robot continues to forage for the remaining pucks. This type of foraging task is considered to be similar to feeding behaviors in nature, if the pucks and robots are assumed to be the prey and predator, respectively.

Robots can forage for both the puck types: red and green pucks; however, each robot can be simultaneously assigned to only one type of task, i.e., collecting either red or green pucks. Each robot has two response threshold values for the two given tasks, i.e., the foraged red and green pucks, respectively, and each robot performs the task of foraging for a specific puck, while maintaining its foraging state.

In the foraging mission, robots may switch their current task according to an individual control policy when it is appropriate to control the balance or improve the overall performance of the system. Here, a task allocation algorithm for regulating the population of agents in proportion to the number of given tasks is considered. That is, the dynamic task allocation maintains the proportion of robots with the tasks of foraging for red and green pucks, respectively, such that they are equal to the proportion of red and green pucks in the arena. Therefore, if 30% of pucks in the foraging arena are red pucks, 30% of the robots should forage for red pucks. The system performance can be determined from the number of foraged pucks, time spent or energy consumed during the completion of a given task. If some costs are incurred for switching a task, it is also recommended that such task switching be minimized while maintaining the desired division of labor.

### 2.2. Robot Behaviors

**Figure 2:**
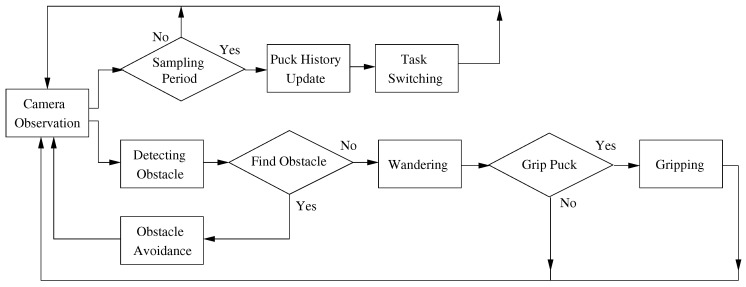
Flowchart of robot behaviors.

The robot behaviors include observation using a camera, avoiding obstacles, wandering, gripping and task switching. Each robot observes the surrounding environment through its own sensors and grips the closest puck within a constant distance. The robot simultaneously detects and avoids obstacles in order to avoid collisions. In addition, the robot changes the foraging target based on the individual control policy. A flowchart of the robot behaviors is shown in Figure 2, and the details of the robot behaviors are explained in the following subsections.

The simulation experiments are implemented using MATLAB program based on a realistic model of the ActivMedia Pioneer 3 DX mobile robot (Adept MobileRobots, Amherst, NH, USA). The sensors data for collision avoidance or motor actions for movement are considered with the inclusion of noisy signals to imitate real systems in which noise is experienced. However, the observation and gripping behaviors in real robots may be slightly different from those in simulated robots. They never fail to grip a puck and obtain exact information using their own sensors. In addition, the delivery of the puck is not considered in the simulation. Therefore, the performance of real swarm robots may be slightly different from that of simulated robots. However, the results may be almost identical with a common simulator, such as that in Gazebo [32] or Swarmanoid [33].

#### 2.2.1. Observation

Each robot has two types of tasks—collecting red pucks or green pucks—and it can switch between tasks depending on the task demand. The robot is equipped with a camera and captures the scene in front of it at $\pm 30^{\circ }$ within a 5 m range. Using this visual information, the robot observes nearby pucks, and it updates its view history of the pucks. All of the robots maintain a limited, constant-sized history that stores the most recently observed puck types. It does not contain the identity number or location of a detected puck. Only the last *N* observed pucks are stored in the puck history and used to estimate the desired global task demand. If there is enough space in the history, all of the information is stored. Otherwise, the oldest data are replaced by the new data. The puck history may be biased based on where the robot is located, and the robot improves the estimation of the task demands by wandering in an arena.

As an observation behavior, the robot stores the puck observation for every 2 m movement. This is an important thing because continuous observation may cause duplicated results in the puck history, which would reduce the calculation accuracy of the global task demand. An exact division of labor may then not be successfully obtained. The maximum forward velocity of the robot is 0.25 m/s; therefore, the robot camera captures an image for every eight wandering steps.

#### 2.2.2. Obstacle Avoidance

Each robot uses eight infrared (IR) sensors to perform the obstacle avoidance behavior. Using IR sensors is effective in the area of autonomous robotics [34,35,36]. Each sensor is equipped on the front side of the robot at a uniform distance from each other to cover 180°, and their detecting range is approximately 0.5 m. The robot changes its current moving direction when the sensors detect obstacles.

If the right sensors detect obstacles, the robot turns to the left; conversely, it turns to the right when the left sensors detect obstacles. The robot turns away from the obstacle at an angle of 45° in either case. If the obstacles are simultaneously detected on both parts, the robot changes its moving direction by turning $180^{\circ }$ in the counterclockwise direction. However, at times, these constant angle changes may lead to similar patterns in the movement direction and cause the occurrence of a congested group of robots if the robots are gathered in a specific area. To avoid this congestion, a Gaussian random variable is included to obtain a variance in the obstacle avoidance angle.

Obstacle avoidance behavior is a basic behavior that is critical to the safety of the robot. Therefore, the occurrence of collisions with other robots and arena boundaries should be eliminated. However, the puck is not considered as an obstacle because because the robot may end up spending considerable time in obstacle avoiding behaviors.

#### 2.2.3. Wandering

The size of the foraging robot is 0.3 m in diameter, and there is no communication between the robots. A robot can move forward and backward at the maximum velocity of 0.25 m/s. Apart from saving information in the puck history, the robot forages for a puck at each time step. The initial movement direction of each robot is randomly set from −π to π, and the robot maintains the moving direction until a desired puck is detected. If a robot finds the pucks of the same color as that of the current robot task, the robot then turns toward the closet puck location and moves ahead to grip it. The robot does not perform wandering behavior if the obstacle avoidance behavior or puck gripping is performed. If the robot senses a puck of a type that is different from its own interest type, it ignores the puck.

#### 2.2.4. Gripping

If no obstacle avoidance occurs and the distance to the desired puck is less than 0.3 m, the robot grips the puck. The gripped puck is immediately removed from the environment. After the puck is removed, a new puck of the same color is placed at an arbitrary location in the arena. The robot then moves to another location to search for another puck. The wandering behavior and obstacle avoidance behavior are not performed during the performance of the gripping behavior.

#### 2.2.5. Task Switching

Each robot should decide the puck color that is to be foraged for in order to regulate the division of labor in a multi-robot system. The individual robot can estimate the approximate global task demand using puck information stored in the history and by measuring the ratio of the observed puck colors. After a robot updates its puck history in an observation behavior, it re-evaluates the task switching function and decides whether it should change its current foraging task or not. There is no communication between robots, and robots only use the information in their own puck history to estimate the task demand. Extra time is required to change the task, and the robots pause at their current location to change their task. Details regarding the proposed task switching function are provided in the next subsection.

## 3. Proposed Method

### 3.1. Modeling

There are *M* robots, and each robot can perform one task from among *N* tasks. The number of robots assigned to task j∈{1,…,N} at time *t* is denoted by $m_{j}\left(t\right)$, and the ratio of robots performing task *j* is defined as $x_{j}\left(t\right)=m_{j}\left(t\right)/M$. If a robot performing task *j* can switch its task to task *k*, the transition rate, $\alpha_{jk}\geq 0$, can be defined to model the task transfer from one task to another. Using the transition rate, the ratio of robots performing task *j* can be represented by the following linear equation:

(1) $$\frac{dx_{j}\left(t\right)}{dt}=\sum_{k=1,k\neq j}^{N}\alpha_{kj}x_{k}\left(t\right)-\sum_{j=1,j\neq k}^{N}\alpha_{jk}x_{j}\left(t\right)$$

Equation (1) represents the average change rate for each task. Using this equation, the steady-state distribution of the group can be predicted across various tasks, and it always converges to the steady state regardless of the choice of $\alpha_{jk}$ [37,38].

### 3.2. Task Switching Algorithm

The desired task distribution can be obtained by appropriately selecting the individual transition rate. Based on the response threshold model, the following task switching function is defined.

(2) $$P_{ij}\left(t\right)=S_{ij}\left(t\right)-\theta_{ij}$$

where $S_{ij}\left(t\right)$ is the estimated global task demand for the *j*-th task of the *i*-th agent at time step *t*, and $\theta_{ij}$ is the response threshold value for the *j*-th task of the *i*-th agent that determines the tendency to perform the corresponding task. For each agent, the score $P_{ij}\left(t\right)$ is calculated using the difference between the task demands and the threshold values for all of the tasks, and the agent chooses the task with the maximum score. If the task demand is increased and the response threshold value is decreased, the calculated value of the score $P_{ij}\left(t\right)$ is high. A robot with a lower threshold starts to perform a task earlier than one with a higher threshold for the same task. This mechanism is represented in the task switching algorithm.

The score value $P_{ij}\left(t\right)$ for the *i*-th agent to work on the *j*-th task at the time step *t* is obtained, and each robot switches the current performing task depending on the result obtained from Equation (2). In order to estimate the global task demand, the robot calculates the proportion of each type of puck by counting the number of red and green pucks in the puck history and estimates the task demand $S_{ij}\left(t\right)$ as follows:

(3) $$S_{ij}\left(t\right)=\frac{1}{L}\sum_{l=1}^{L}{color}_{ij}^{l}\left(t\right)$$

where ${color}_{ij}^{l}\left(t\right)$ is the color *j* in the *l*-th puck history of robot *i* at time *t*. If the *l*-th puck color is color *j*, then ${color}_{ij}^{l}=1$, otherwise, it is zero. *L* is the length of the puck history and is set to L=20.

The task demand is estimated using the moving average of the color scores in the puck history. The moving average is commonly applied to the time series data to smooth out short-term fluctuations and read long-term trends. Conceptually, if the length of the puck history increases, the changes in the task demand, as well as the frequency of task switching may decrease. Therefore, an improved approach is proposed as follows:

(4) $$S_{ij}^{\prime }\left(t\right)=\frac{1}{L}\sum_{k=0}^{L-1}S_{ij}\left(t-k\right)=\frac{1}{L}\frac{1}{L}\sum_{k=0}^{L-1}\sum_{l=1}^{L}{color}_{ij}^{l}\left(t-k\right)$$

The first method in Equation (3) (called History1) uses the moving average of the puck history; the second method in Equation (4) (called History2) uses the moving average of the estimated task demand obtained from the first method. This method enlarges the length of the task history to 2*L*.

Each robot has an equal number of response threshold values for the given tasks. In the foraging task, each robot has two types of thresholds for foraging red and green pucks, respectively. In the response threshold model, the division of labor can be regulated depending on the distribution of the response threshold values in the group. In previous works, the effects of randomly selected threshold values [39] and a single threshold value for sequentially ordered tasks [40] were studied. In the basic concept of the response threshold model, a single threshold value is required for a corresponding task. To apply a single threshold for two tasks, the task should be ordered sequentially, proportional to its task demand, and we would require a different type of task selection function. In the case of two tasks, the performance of a single threshold can be the same as those of the multiple thresholds. However, for more than two tasks, the performance is very different because the task can be only changed to the before or next task. In this paper, an individual robot has a constant response threshold value, which can be presented as follows:

(5) $$\theta_{i,red}=\frac{1}{M-1}\times \left(i-1\right),\;i=1,...,M$$

(6) $$\theta_{i,green}=1-\frac{1}{M-1}\times \left(i-1\right),\;i=1,...,M$$

where *M* is the total number of robots. The assigned response threshold values for each task can be between zero and one. The sum of the response threshold values for each robot is one. Here, we require an individual agent to have response threshold values at evenly-spaced intervals within the range from the minimum to the maximum value. Subsequently, we shall compare the proposed distribution of the response threshold values with the randomly selected values [39].

Overall, in the case of a system, the task transition rate $\alpha_{jk}\left(t\right)$ in Equation (1) can be defined as:

(7) $$\alpha_{jk}\left(t\right)=\frac{m_{jk}\left(t\right)}{m_{j}\left(t\right)}$$

where $m_{jk}\left(t\right)$ is the number of robots that are currently performing task *j* and have the maximum scores for performing task *k*. This value changes depending on the estimated task demand $S_{ij}\left(t\right)$ and the response threshold value $\theta_{ij}$.

Based on the response threshold model, the individual response for the same task demand varies according to the individual response threshold value for each robot; this method used for selecting a specific task is a specialization for a specific task The agents with low threshold values tend to specialize in the corresponding task. The smaller the threshold value is for a given specific task, the greater is the activation achieved for that task. Therefore, the task specialization for a division of labor is well demonstrated using this model. This specialization tendency can also reduce the number of task switches. We assume that there is a cost incurred for task switching, time consumption, and the minimization of such task changes would be advantageous for group behavior.

## 4. Experimental Results

To analyze the proposed algorithm in the foraging task, not only the total count of foraged pucks, but also the total occurrences of task switching for all of the robots are measured. Fifty pucks were randomly distributed in an arena, and twenty robots continuously moved from one place to another to forage for pucks. At the beginning, half of the robots were tasked with foraging for a red puck, while the others were tasked with foraging for a green puck. For each experiment, 20 independent runs were averaged, and the results with *History1* and *History2* were compared to a ratio model (called Ratio) that was presented in the study by Jones and Mataric (2003) [26].

### 4.1. Result with Changes in Task Demands

**Figure 3:**
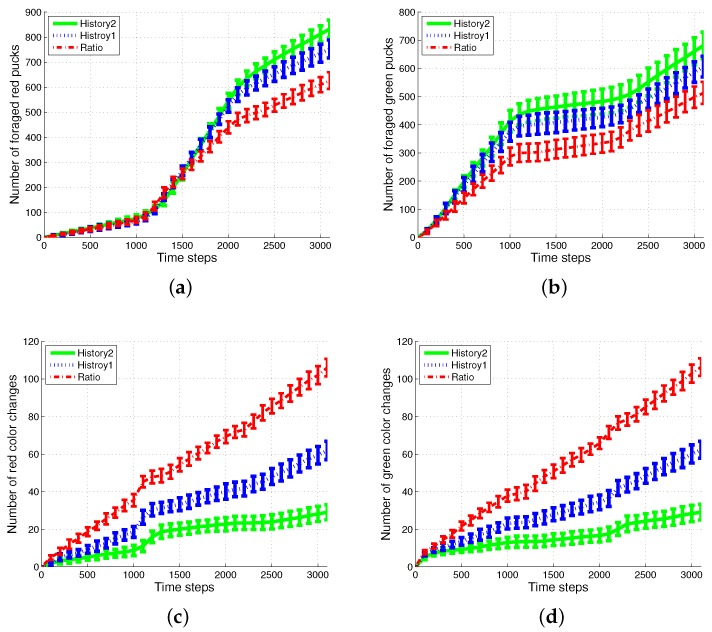
Number of foraged (**a**) red and (**b**) green pucks and occurrences of task switching in foraging for (**c**) red and (**d**) green pucks.

First, the basic situation in which the ratio of pucks was changed in the time course was used as a test condition. During the first 1000 simulation time steps, the number of red pucks was maintained at 30%. That is, there were 35 green pucks and 15 red pucks in a given arena. After the first 1000 time steps, the number of red pucks was switched to 80% and 50% for the next two consecutive sets of 1000 simulation time steps, respectively. The results of the simulations during the aforementioned 3000 simulation time steps are shown in Figure 3. The red dashed-dotted line represents the performance of the ratio model; the other lines represent the results obtained on using the response threshold model. The blue dotted line represents *History1*; the green solid line represents *History2*. The error bar shows the standard deviation over 20 runs.

In all of the methods used, each type of puck—red and green—was steadily collected as shown in Figure 3a,b. However, the greatest number of pucks was foraged in *History2*. This means that robots could spend more time in collecting pucks, which would reduce the time wasted. As shown in Figure 3c,d, in terms of reducing the task switching, the response threshold model, especially *History2*, showed a greater improvement than the result of *Ratio*. The frequent task changes lead to the poor foraging performance of the ratio model.

**Figure 4:**
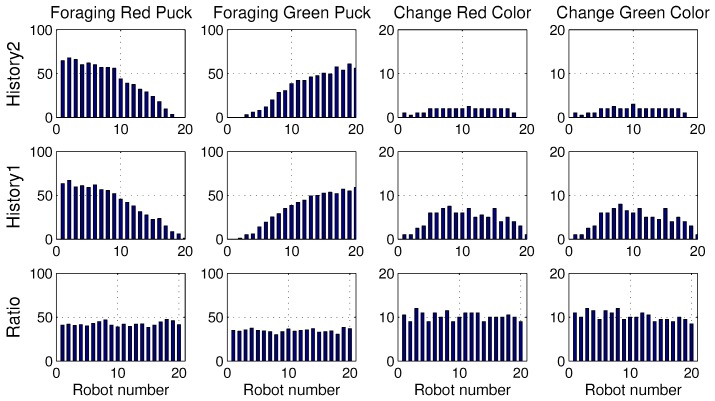
Number of foraged pucks and task changes of an individual robot.

These results are obtained owing to the specialization tendency of each robot. If some robots start to intensively forage for one specific puck, those robots had a tendency to forage for pucks of the same color. This tendency is demonstrated well in Figure 4, which displays the number of pucks foraged by an individual robot. The first row presents the results of *History2*. Each row shows the foraged red pucks, foraged green pucks, task changes for the red pucks and task changes for the green pucks in the left-to-right direction.

In the ratio model, all of the robots foraged for red and green pucks in a similar manner. However, in the response threshold model, the pucks were selectively foraged for by each robot. Some robots were strongly specialized in a specific task. From the results, it can be observed that Robots 1 and 2 only foraged for red pucks and Robots 19 and 20 preferentially foraged for green pucks. Accordingly, there were few task changes for another type of puck in these robots. However, other robots were softly specialized depending on a slight gap between the response threshold values of the two tasks, and they foraged for both types of pucks. Therefore, the majority of the task changes occurred for these robots. We often observe that time and energy are required for task changes in real application problems. Minimizing the task changes would reduce this time consumption, and thus, *History2* may be a preferable solution.

**Figure 5:**
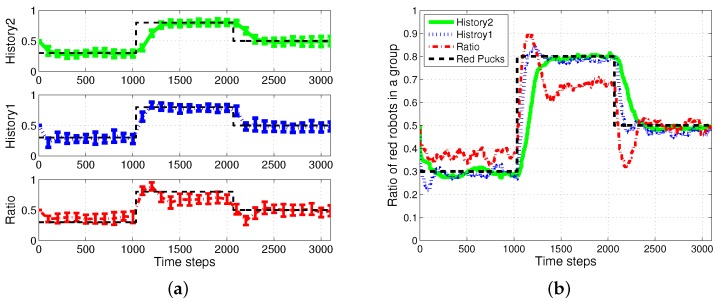
Ratio of robots foraging for red pucks in a group. (**a**) Individual trend of each algorithm; (**b**) overlapping trends of all algorithms.

Figure 5 shows the changes in the ratio of robots foraging for red pucks in each algorithm. Both *History1* and *History2* show the same ratio of robots foraging for red pucks in a group as the ratio of red pucks. History1 shows a faster convergence tendency; however, it shows greater overshooting than History2. In general, the shorter puck history resulted in a quicker convergence to the desired state; however, more frequent task changes are required for robots in a group than for individual robots.

The *ratio* model showed the quickest reaction to the task changes for red pucks. Nevertheless, it showed the worst performance in terms of overshooting and some errors in the ratio of robots foraging for red pucks. In the ratio model, a robot focused on balancing the estimated task demand and the ratio of the foraging task performed by neighboring robots. In the case in which the proportion of one specific task was given, a few extra robots were assigned to forage for the minor-color pucks. This feature produced a better result in the foraged pucks with a minor proportion in the whole population of pucks. In the two cases in which the ratios of the red pucks were 30% and 80%, respectively, there were more robots foraging for minor pucks than the ratio of minor pucks, and this tendency caused some gap between the global task demand (portion of two types of pucks) and the assigned robots in the *ratio* model.

**Figure 6:**
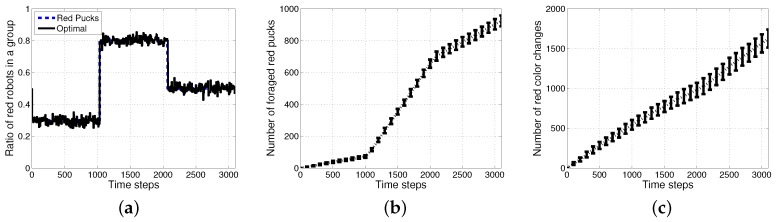
Result of an optimal method using a stochastic approach. (**a**) Ratio of red robots in a group; (**b**) number of foraged red pucks; (**c**) number of task changes in foraging for red pucks.

Figure 6 shows the results of an optimal method with the assumption that all of the agents knew the exact ratio of each puck. In this case, an individual robot could easily select its task using a stochastic strategy and select a task probabilistically in proportion to the ratio of tasks. Further improved performances can then be obtained in the aspects of accuracy in the ratio of red robots and the number of foraged red pucks as shown in Figure 6a,b. However, there is a requirement for more than tens of times the task switches as shown in Figure 6c.

**Figure 7:**
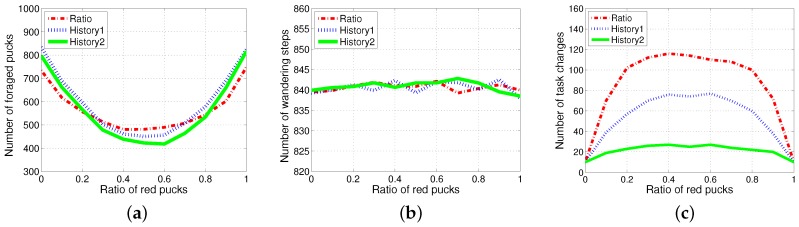
Performance evaluation with varying ratios of red pucks. (**a**) Number of all foraged pucks; (**b**) number of wandering steps; (**c**) number of task changes.

An individual robot has to exert energy to forage for pucks and to grip the pucks. In addition to these costs, some extra costs were incurred due to the task switching. To observe the effect of task switching, the three methods, *History1, History2* and *ratio*, were evaluated for varying ratios of red pucks for 1000 simulation time steps with no additional time for the task change, but only counting the number of task changes. The performance in the collection of pucks changed depending on the ratio of foraging tasks. The total number of foraged pucks and wandering steps was almost the same for the three algorithms, as shown in Figure 7a,b. Therefore, the energy required for wandering and gripping did not differ across these cases; moreover, the total consumed energy mainly depended on the number of task switches for each experiment. *History2* showed an improved performance in the aspect of the task changes as shown in Figure 7c. The results for the two methods History1 and History2 compared with those for the ratio model in terms of change in demand were shown. The response threshold models used a fixed constant-sized history for storing the most recently observed puck types. History2 exhibits a better performance than History1 in terms of task switching. This induced an improved performance in the number of foraged pucks, while it probably guessed the global task demand (the current proportion of pucks in the environment).

Therefore, the difference in the performances between the ratio model and the response threshold model was evident when there were costs related to task switching. In a constraint condition with a cost for task switching, if the cost of task switching increased, the total energy wasted in the foraging for pucks increased rapidly. Task switching occurred infrequently in the response threshold model as compared to the ratio model. Thus, the response threshold model is more suitable if the task switching cost is high.

### 4.2. Results with Changes in the Size of Puck History

**Figure 8:**
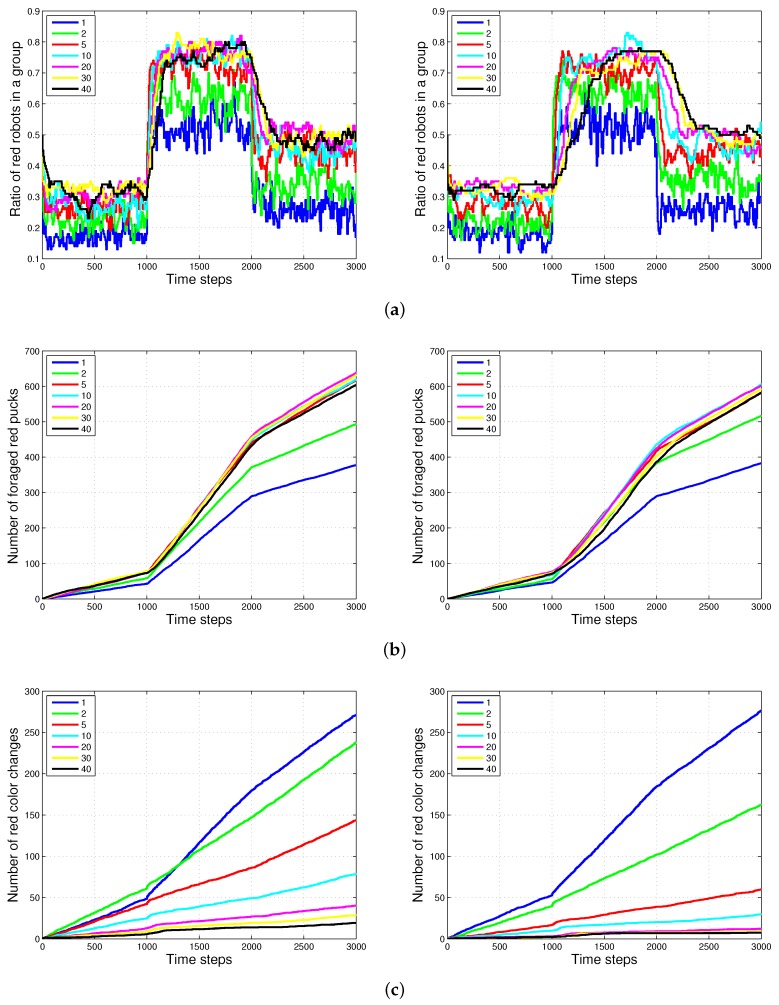
Performance of the *History1* and *History2* methods with various lengths of the puck history. The various colors indicate varying sizes of the puck history from L=1 to L=40. (**a**) Ratio of red robots in a group in History1 (left) and History2 (right); (**b**) number of foraged red pucks in History1 (left) and History2 (right); (**c**) number of task changes for red pucks in History1 (left) and History2 (right).

Figure 8 shows the experimental results of the two methods, History1 and History2 for varying sizes of the puck history. For the two methods, as the length of the puck history increases, the accuracy of the proportion of robots foraging for red pucks in a group increases. The number of robots collecting red pucks converged to the desired results; however, the total number of task changes decreases as the length of the task queue increases. We also observed that the time required to converge to the ratio of robots to forage for red pucks increases with the History2 method. This effect is the result of the specialized tendency of History2.

**Figure 9:**
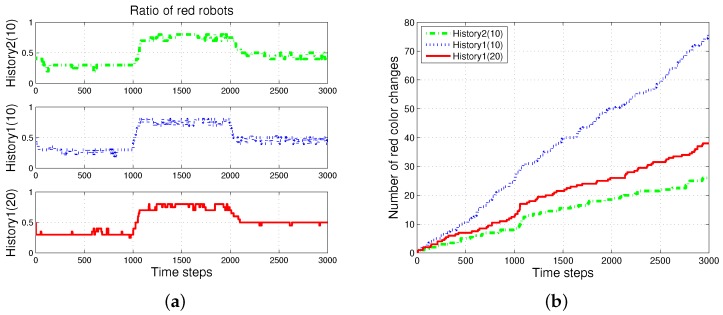
Comparison results of History1 (L=10), History1 (L=20) and History2 (L=10). The numbers in parentheses refer to the length of the puck history. (**a**) Ratio of red robots in a group; (**b**) number of red color changes.

Generally, the total number of task switches decreases when the size of the task queue increases. When the two methods with the same size of task queue were compared, History2 was found to be more effective than History1 (see History1 with L=10 and L=20 and History2 with L=10 in Figure 9). As the size of the task queue with History1 increases to twice the original value (from L=10 to L=20), the ratio of red robots becomes more accurate, i.e., even better than that in History2 (L=10). Actually, History2 uses double the size of the task queue used in History1 for L=10 in Equation (3). However, the number of task switches for History2 (L=10) is still less than that for History1 (L=20). The moving average of the puck history has a positive effect on the task changes.

**Figure 10:**
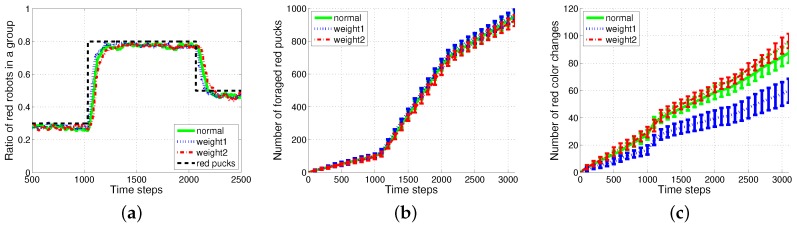
Results of weighted History1 (L=10). (**a**) Ratio of red robots in a group; (**b**) number of foraged red pucks; (**c**) number of red color changes.

Figure 10 shows the results of the weighted History1 (L=10); the green solid line represents the original History1, the blue dotted line represents the weighted History1 where the weight is set to $\left[1^{2},2^{2},...,{\left(L-1\right)}^{2},L^{2}\right]$ to emphasize the newest history; and the red dashed-dotted line represents the reversely weighted History2. Owing to the weight of the newest history (weight1), a slightly faster response to the change in task demand and improved performance in terms of smaller task changes were observed with the appropriate proportion of pucks to the task demand than the original History1.

### 4.3. Results with Changes in Vision Sampling Period

**Figure 11:**
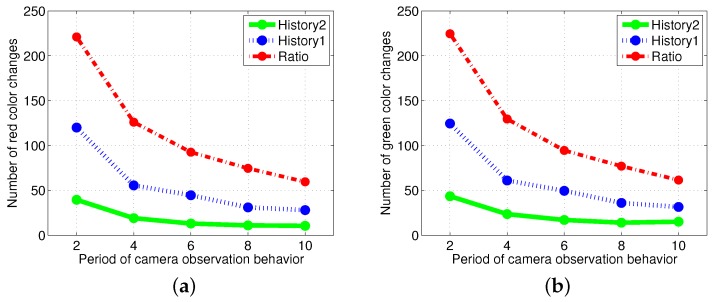
Comparison of the results of the number of task changes with a variation in the vision sampling period from two to ten time steps. (**a**) Task changes for red pucks; (**b**) task changes for green pucks.

To avoid recording the same puck repetitively in the puck history, the robots sense the environment once every eight time steps using visual information. Figure 11 shows that increasingly frequent vision sensing leads to increased task changes. In addition, the performance of the response threshold model was more robust for the change in the vision sampling period than in the ratio model.

### 4.4. Results with a Fixed Number of Tasks

**Figure 12:**
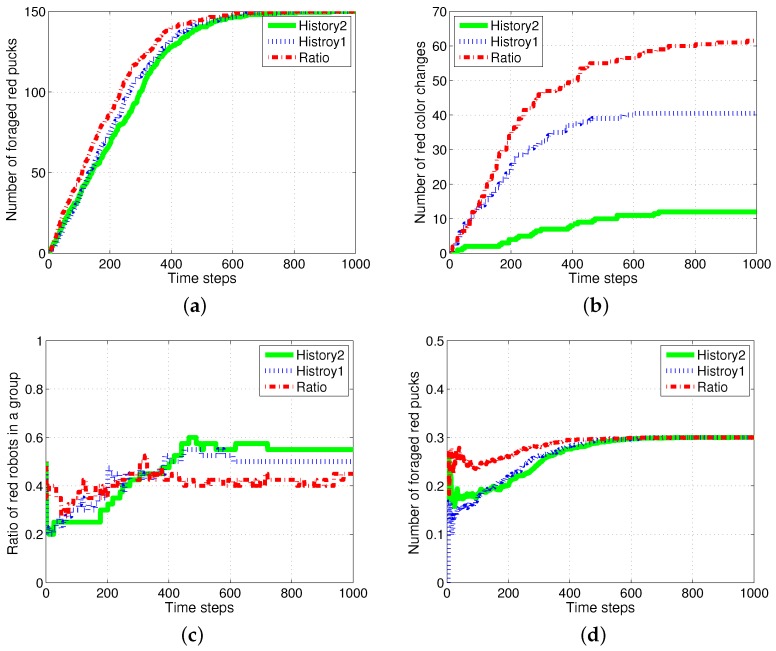
Results of foraging tasks when the foraged pucks were not reproduced. (**a**) Number of foraged red pucks; (**b**) number of task switches in foraging for red pucks; (**c**) ratio of robots foraging for red pucks; (**d**) ratio of foraged red pucks.

The case in which the foraged pucks are not reproduced was considered, and Figure 12 shows the results. There were 500 pucks in the same arena; 20 robots wandered in order to forage for the pucks. The proportion of red pucks was set to 30% at the starting of the simulation. The foraged pucks were not reproduced in the arena. Therefore, the total number of pucks decreased as time passed. In this foraging task, the division of labor results differed minimally from those of the ratio model.

The red and green pucks were steadily foraged in both the models, as shown in Figure 12a. The number of task switches was smaller in the response threshold model, as shown in Figure 12b. This is similar to the results obtained in the previous experiments. However, several robots switched their tasks to forage for green pucks at an earlier period. The green pucks were preferentially foraged because the ratio of green pucks was higher than that of red pucks. After some simulation time steps, the robots began to switch their states to forage for red pucks according to the increasing task demand for foraging for red pucks. Therefore, the ratio of robots foraging for red pucks and foraged red pucks changed, as shown in Figure 12c,d. These features are largely shown in the response threshold model, especially in History2, which had a strong specialization characteristic.

### 4.5. Results with Changes of Threshold Distribution

**Figure 13:**
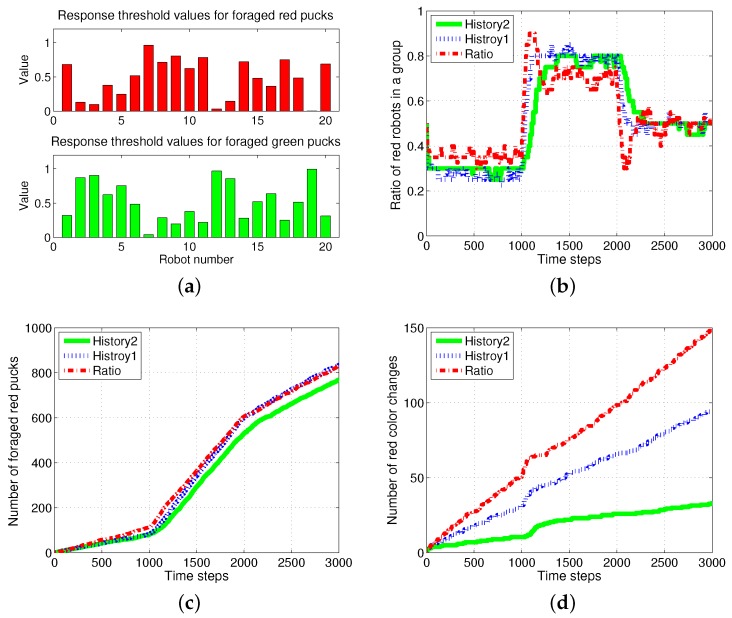
Randomly distributed pattern for response threshold values for two foraging tasks. (**a**) Response threshold value in a random pattern; (**b**) ratio of robots for red pucks in a group; (**c**) number of foraged red pucks; (**d**) number of task switches for red pucks.

It is necessary to determine the appropriate response threshold value to improve the system performance in the response threshold model. In the study of [41], the response threshold model was implemented using artificial neural networks. In the proposed approach, the response threshold values in the group of robots were at evenly-spaced intervals within the range from the minimum to the maximum value. If randomly assigned values were used, as shown in Figure 13a, a slightly decreased performance in terms of the ratio of the division of labor in the group was obtained, as shown in Figure 13b. These performances are of the same foraging task used in Figure 3. A similar improved performance in the rate of foraged pucks as compared to the ratio model was obtained. In addition, the number of task switches was markedly improved in comparison to the ratio model, as shown in Figure 13c,d.

### 4.6. Drawback of Specialization in the Foraging Task

Despite the previous results, the proposed method based on the response threshold model can be effective in all aspects. If the proportion of foraged pucks is considered as the index of the system performance, the response threshold model showed a worse result than the ratio model. A robot in a response threshold model has a specialized tendency to perform one specific task. Thus, specialized robots should move a much greater distance to forage for specific color pucks without task switching. However, if the proportion of some pucks is much smaller than those of other colors, the probability of obtaining the specific puck is reduced, and the minor pucks have a decreased chance of being foraged by the robots than in the ratio model.

In fact, in a circular arena, the probability that the robots will forage for specific pucks is related to the square of the ratio of the task. It the portion of pucks is 30% and 70% for red and green pucks, respectively, the chances that the robots with the task of foraging for red pucks will find the red pucks may be $0.3^{2}/\left(0.3^{2}+0.7^{2}\right)*100\%$. The probability of obtaining a foraged red puck may be 15.5%.

**Figure 14:**
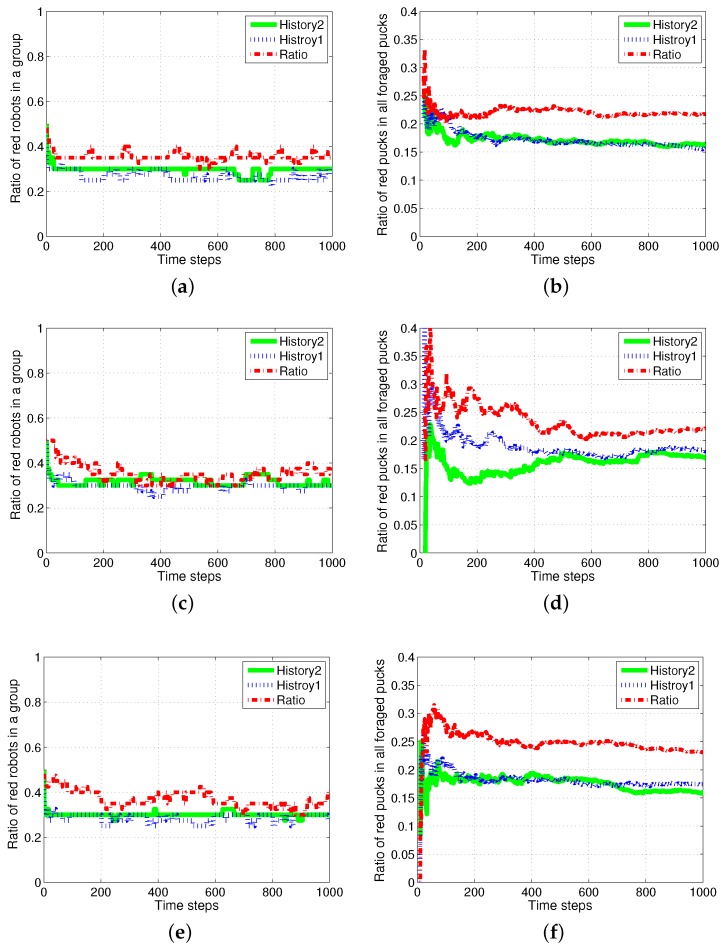
Ratio of robots foraging for red pucks in a group and the ratio of red pucks to all foraged pucks. (**a**) Robots foraging for red pucks (front $60^{\circ }$); (**b**) ratio of red pucks (front $60^{\circ }$); (**c**) robots foraging for red pucks (front $20^{\circ }$); (**d**) ratio of red pucks (front $20^{\circ }$); (**e**) robots for red pucks (front $20^{\circ }$, triple pucks); (**f**) ratio of red pucks (front $20^{\circ }$, triple pucks).

Figure 14a–f show the results with some changes in the experimental settings when the proportion of red pucks was fixed to 30% during 1000 simulation time steps; (a) and (b) show the results with the vision camera angle $60^{\circ }$ from the front; (c) and (d) ratio show the results with the vision camera angle $20^{\circ }$ from the front; and (e) and (f) show the results with the vision camera angle $20^{\circ }$ from the front; the number of pucks increased three times. Figure 14a,b shows the ratio of robots foraging for red pucks and the foraged red pucks. Although the ratio of robots in the group matched the ratio of tasks, the ratio of foraged pucks was lower for the response threshold model, as expected. Therefore, if the ratio of foraged pucks is regarded as the system performance measure, the response threshold model showed a weakness because there exists a trade-off between the accuracy and the number of foraged minor pucks.

If the robots capture images of the front with a narrow angle, the exact estimation of the global task demand will be difficult, and the results of the division of labor will decrease in accuracy and will require more time to become stable, as shown in Figure 14c,d. However, when the density of the distributed pucks increased by three times that of the original experiment, despite the poor camera detection ability, the performance of the division of labor increased in accuracy, as shown in Figure 14e,f. In a foraging task in a circular arena, the results obtained with various systems are related with not only the ability of the robots, but also the surrounding environments.

Additionally, the obstacle avoiding behavior and distribution of pucks in a circular arena make it difficult to search for specific pucks. Here, pucks are not considered as obstacles, and robots can pass by the pucks easily. If the pucks are considered as obstacles, the movements of the robots in a fixed area become restricted. The inaccuracy in the estimation of task demand and frequent obstacle avoidance may limit the specialization tendency in the response threshold model.

**Figure 15:**
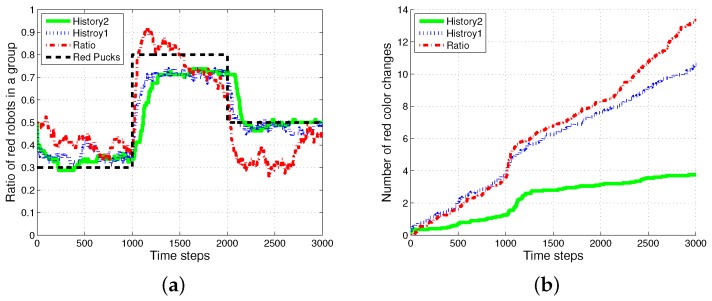
Ratio of robots foraging for red pucks in a group, and the number of task switches for red pucks. (**a**) Robots for red pucks in a group; (**b**) number of task switches for red pucks.

We tested another experiment in which robots treated the pucks as obstacles. Figure 15 shows the ratio of robots foraging for red pucks in a group, and the number of task switches for red pucks when the pucks are considered as obstacles. These performances are evaluated for the same foraging task as used in Figure 3 and Figure 5; however, pucks are considered as obstacles. We found a slight mismatch between the global task demand and the ratio of foraged robots in both *History1* and *History2*, as shown in Figure 15a, and the number of task changes for red pucks was similar to the result obtained with *ratio*, as shown in Figure 15b. However, *History2* still showed the best performance in terms of reducing the task changes among the three algorithms.

## 5. Conclusions

In this paper, a response threshold model is applied for improving the performance of a foraging task. We suggest a method that includes the use of a puck history to estimate the global task demand. Each robot estimates the desired task demand using the moving average of the observed pucks in the course of time. The robots are characterized by their own response threshold values. The threshold values are uniformly distributed among a set of robots to allow for the diversity of the response characteristics.

The division of labor generally uses a global task demand as an important variable. Based on the various experiments, an appropriate choice that uses the puck history of the recently-observed pucks can be used as a guide in a multi-robot system. Interestingly, local information regarding the surrounding environment can be used to estimate the global task demand well. Each robot has a specialization tendency, and the overall system thus has the advantage of greatly reducing the occurrence of task changes. If there is an extra cost for task switching, the system based on the response threshold model may greatly improve the energy efficiency by reducing the task switching frequencies.

The foraging task used for the tests involves exploration for pucks, and the task switching of the robots seems inevitable in this type of task. The suggested method has the effect of reducing the task switches by inducing the division of labor. In future work, the behaviors of robots will be investigated using a Swarmanoid robots simulator, and the proposed model will be used in various applications to analyze the performance of real robots. Furthermore, a more appropriate method for improving the system performance will be studied.
